# Understanding Pediatric Bipolar Disorder Through the Investigation of Clinical, Neuroanatomic, Neurophysiological and Neurocognitive Dimensions: A Pilot Study

**DOI:** 10.3390/brainsci15020152

**Published:** 2025-02-03

**Authors:** Alessio Simonetti, Evelina Bernardi, Sherin Kurian, Antonio Restaino, Claudia Calderoni, Emanuela De Chiara, Francesca Bardi, Gabriele Sani, Jair C. Soares, Kirti Saxena

**Affiliations:** 1 Department of Psychiatry and Behavioral Sciences, Baylor College of Medicine, Houston, TX 77030, USA; alessio.simonetti@policlinicogemelli.it (A.S.); sherin.kurian@bcm.edu (S.K.); kirti.saxena@bcm.edu (K.S.); 2 Department of Neuroscience, Head-Neck and Chest, Section of Psychiatry, Fondazione Policlinico Universitario Agostino Gemelli IRCCS, 00168 Rome, Italy; 3 Department of Neuroscience, Head-Neck and Chest, Section of Psychiatry, Università Cattolica del Sacro Cuore, 00168 Rome, Italy; evelinabernardi@gmail.com (E.B.); restainoantonio11@gmail.com (A.R.); claudia.calderoni1991@gmail.com (C.C.); dechiaraemanuela@gmail.com (E.D.C.); francesca.bardi97@gmail.com (F.B.); 4 Department of Child and Adolescent Psychiatry, Texas Children’s Hospital, Houston, TX 77054, USA; 5 Department of Psychiatry and Behavioral Sciences, University of Texas Health Science Center, Houston, TX 77030, USA; jair.c.soares@uth.tmc.edu

**Keywords:** pediatric bipolar disorder, behavior, cognition, emotion, magnetic resonance imaging, electroencephalography

## Abstract

**Background**: Pathophysiological models of pediatric bipolar disorder (PBD) are lacking. Multimodal approaches may provide a comprehensive description of the complex relationship between the brain and behavior. **Aim**: To assess behavioral, neuropsychological, neurophysiological, and neuroanatomical alterations in youth with PBD. **Methods**: Subjects with PBD (n = 23) and healthy controls (HCs, n = 23) underwent (a) clinical assessments encompassing the severity of psychiatric symptoms, (b) neuropsychological evaluation, (c) analyses of event-related potentials (related to the passive viewing of fearful, neutral, and happy faces during electroencephalography recording, and (d) cortical thickness and deep gray matter volume measurement using magnetic resonance imaging. Canonical correlation analyses were used to assess the relationships between these dimensions. **Results**: Youth with PBD had higher levels of anxiety (*p* < 0.001) and borderline personality features (*p* < 0.001), greater commission errors for negative stimuli (*p* = 0.003), delayed deliberation time (*p* < 0.001), and smaller risk adjustment scores (*p* = 0.002) than HCs. Furthermore, they showed cortical thinning in the frontal, parietal, and occipital areas (all *p* < 0.001) and greater P300 for happy faces (*p* = 0.29). In youth with PBD, cortical thickening and P300 amplitude positively correlated with more commission errors for negative stimuli, longer deliberation times, reduced risk adjustment, higher levels of panic and separation anxiety, and greater levels of negative relationships, whereas they negatively correlated with levels of depression (overall loadings > or <0.3). **Limitations:** Small sample size, cross-sectional design, and limited variables investigated. **Conclusions**: This preliminary work showed that multimodal assessment might be a viable tool for providing a pathophysiological model that unifies brain and behavioral alterations in youth with PBD.

## 1. Introduction

Pediatric bipolar disorder (PBD) is a severe illness with unique features when compared to adult-onset bipolar disorder, including differences in symptom manifestation, treatment efficacy, severity, duration, and frequency of hospitalizations [1]. PBD is characterized by cyclic mood alterations and is often associated with emotional lability, anxiety [2], and aggressive, risky, or hostile behaviors [3]. These traits frequently lead to interpersonal difficulties and family problems [1]. Altered behavior and pathological traits in youth with PBD are accompanied by subtle neurocognitive dysfunction related to affective processing [4], gratification response [5], working memory [5], decision-making [6], attention [7], and problem-solving [8]. Notably, the most frequently affected areas include impulsivity [6], decision-making challenges [9], and difficulties in managing negative events [10]. In the last 20 years, substantial efforts have been made to define the neural basis underlying these alterations to facilitate early diagnosis and develop tailored treatment plans for individuals with PBD [11]. Recent advances in functional and structural magnetic resonance imaging (MRI) [12] and electroencephalography (EEG) techniques [13] have helped researchers define the areas and brain networks involved [14,15]. Areas receiving greater attention include the thalamus [16], hippocampus [17,18], and amygdala [19,20], all of which were discovered to be smaller in youth with PBD as compared to healthy controls (HCs).

Alterations in cortical thickness have also been observed, most of which are associated with deficits in emotional and cognitive control [21]. The most frequent cortical thickenings were identified in the frontal, parietal, and temporal regions, such as the orbitofrontal cortex [22], anterior cingulate cortex [23], insular cortex [24], supramarginal gyrus [21,25,26], and orbitofrontal cortex [27].

EEG techniques employ event-related potentials (ERPs) and enrich the knowledge gained by MRI techniques by providing the exact timeframe in which the aforementioned cognitive and emotional alterations occur [28]. Particularly, alterations in the P300 have been shown to reliably detect abnormalities in both cognitive and emotional domains, as well as related behavioral dysregulation [29]. P300 represents a positive deflection observed within the parietal cortex in response to infrequent and salient stimuli [30,31] and is intricately linked with cognitive functions, such as working memory, allocation of attention, and decision-making [32]. Moreover, P300 is associated with circuits related to reward frustration [14] and emotion recognition [33]. A reduced P300 amplitude observed during the recognition of emotions conveyed through facial expressions (happiness, sadness, fear, disgust, and neutrality) has been associated with PBD [34].

To date, studies combining behavioral and biological evaluations in youth with PBD have been conducted [3,4,12,31]. Evidence brought so far suggests that behavioral alterations in youth with PBD are driven by a complex array of neural alterations involving cortical and subcortical areas. Nevertheless, a comprehensive evaluation investigating the psychopathological, neuropsychological, neuroanatomical, and neurophysiological domains is still lacking. Such an approach might ease the definition of a comprehensive physiopathological model of PBD in youth.

Therefore, this study serves as a preliminary work aimed at proposing a cohesive model that establishes connections between behavioral, neurocognitive, neurophysiological, and neurobiological parameters.

The aims of this study are: (i) to identify significant behavioral and neurocognitive alterations in youth with PBD; (ii) to explore neuroanatomical and neurophysiological alterations in this population; and (iii) to evaluate the relationship among all these domains.

We hypothesize that alterations in neurocognitive and behavioral domains, i.e., greater levels of depression, excitement, anxiety, hostility, greater interpersonal and family issues, enhanced response to negative stimuli and rewards, prolonged decision-making times, increased impulsivity and difficulties in assessing risk in participants with PBD as compared to HC. Furthermore, we anticipate P300 reductions in amplitude and increases in latency during emotion recognition and decreased cortical thickness in the frontal, temporal, and parietal networks. Regarding the relationship between cortical thickness and neurocognition, we expect a positive correlation between P300 reduction and cortical thinning in the areas discussed above, as well as an increased number of errors when identifying emotional stimuli and prolonged decision-making times. Conversely, we expect a negative correlation between these neurophysiological and neuroanatomical alterations and difficulties in assessing risk. In terms of behavior, we expect that cortical thickening/reduced volumes in the areas described above correlate with the severity of depression and anxiety disorders, levels of hostility, and negative relationships.

## 2. Materials and Methods

### 2.1. Study Participants

Subjects were recruited from the child and adolescent psychiatric clinic of Baylor College of Medicine in Houston, TX, USA, whereas HC were recruited through advertisements. Written informed consent was obtained from the subjects and their parents prior to enrollment. Inclusion criteria were the following: (i) age between 7 and 17 years; (ii) capability of subjects’ parents to sign a written informed consent form; (iii)comprehension of the English language; (iv) subjects with PBD additionally required a prior diagnosis of bipolar disorder (BD) type I, BD type II, or BD not otherwise specified (PBD-NOS). Exclusion criteria were (i) substance use disorder; (ii) intellectual disability; (iii) autism spectrum disorder; (iv) severe neurological conditions; (v) subject and subject’s parent’s inability to sign a written informed consent; (vi) subject’s unawareness of his/her psychiatric disorder (only for youth with PBD); and (vii) impossibility to understand English language, written or spoken. The diagnoses of BDtype I and BDtype II were established according to DSM-5 criteria; the diagnosis of PBD-NOS was based on the Course and Outcome of Bipolar Youth (COBY) research criteria [34].

### 2.2. Clinical Assessments

Study participants were assessed using (i) the Mini International Neuropsychiatric Interview-KID (MINI-KID) and MINI-KID parent version [35] to determine psychiatric diagnoses and (ii) the Wechsler Abbreviated Scale of Intelligence-II (WASI-II) [36] to determine age- and sex-corrected general intelligence (composite intelligence quotient [IQ] score). The assessment was performed by a trained clinician.

### 2.3. Cortical Thickness

Structural scans using three-dimensional magnetization-prepared rapid gradient-echo imaging (3D MP-RAGE) [37] were obtained on a 3.0 T Siemens Trio scanner with the following parameters: TR = 2500 ms, TE = 3.5 ms, Bandwidth = 190 Hz/Px, field of view = 256 × 256 mm, flip angle = 8°, and voxel size = 1.0 × 1.0 × 1.0 mm. All T1-weighted scans underwent visual inspection to confirm the absence of significant artifacts and were subsequently processed using the FreeSurfer software version 6.0 [38,39]. Cortical thickness measurements were automatically extracted through segmentation of the following regions: left and right banks of the superior temporal sulcus, left and right caudal anterior cingulate, left and right caudal middle frontal, left and right cuneus, left and right entorhinal, left and right fusiform, left and right inferior parietal, left and right inferior temporal, left and right isthmus cingulate, left and right lateral occipital, left and right lateral orbitofrontal, left and right lingual, left and right medial orbitofrontal, left and right middle temporal left and right parahippocampal, left and right paracentral, left and right pars opercularis, left and right pars orbitalis, left and right pars triangularis, left and right pericalcarine, left and right postcentral, left and right posterior cingulate, left and right precentral, left and right precentral general, left and right precuneus, left and right rostral anterior cingulate, left and right rostral middle frontal, left and right superior frontal, left and right superior parietal, left and right superior temporal, left and right supramarginal, left and right frontal pole, left and right temporal pole, left and right transverse temporal, and left and right insula.

### 2.4. Deep Gray Matter Structures

Deep gray matter volumes were estimated using the automated procedure for volumetric measurements of brain structures implemented in FreeSurfer 6.0 [38], running on a CentOS Linux 6.4 system. This automated deep gray matter volume segmentation method has been previously described [40]. In summary, the procedure involves automatic segmentation and labeling of anatomical structures based on an atlas containing probabilistic information regarding structural locations. Each voxel in the MRI volume is assigned a neuroanatomical label using probabilistic data derived from a manually labeled training set. The process begins with an affine registration to Talairach space, followed by initial volumetric labeling and correction for intensity variations due to “intensity field bias.” A high-dimensional, non-linear volumetric alignment to the Talairach atlas is then performed, and the final labeling is completed. This labeling process utilizes an algorithm that combines a subject-independent probabilistic atlas with subject-specific measured values. It assigns values at each point in space based on three types of probabilities: (1) the likelihood that a given point belongs to each label class, (2) the likelihood of a point belonging to a label class based on neighboring points, and (3) the probability distribution function of the measured intensity values at each voxel, estimated separately for each class [40,41]. This method provides results comparable to those of manual region-of-interest (ROI) delineation [42,43] while eliminating biases and delivering anatomically accurate regional volume measurements [40]. To account for individual differences in brain size, intracranial volume (ICV), which includes brain tissue, meninges, and cerebrospinal fluid, was calculated to adjust for regional brain volume analyses [44]. Specifically, the volumes of the deep gray matter structures were corrected for ICV using the proportion method [45].

### 2.5. Event-Related Potentials

Raw EEG signals were recorded using BrainVision Recorder (Brain Products GmbH, Zeppelinstraße 7, Gilching, Germany) from 32 active electrodes placed at locations based on the 10–20 international system of EEG electrode placement (ActiCAP; Easycap GmbH, Zeppelinstraße 7, Gilching, Germany). AFz served as ground; FCz served as reference. The impedances were less than 20 kΩ. Signals were sampled at 250 Hz, filtered between 0.1–1000 Hz, and amplified with a BrainAmp Standard amplifier (Brain Products GmbH, Zeppelinstraße 7, Gilching, Germany). The signals were processed offline using EEGLAB [46] and ERPLAB [47]. Raw data were re-referenced to TP9/10, segmented into epochs ranging from 200 ms before to 1500 ms after the face stimuli, band-pass filtered between 0.1–30 Hz, and baseline corrected. Trials containing incidental, non-repetitive artifacts (e.g., occasional movement artifacts or clipping) were manually excluded prior to conducting independent component analysis (ICA; runica option in EEGLAB) [48]. Artifact-related ICA components (such as eye blinks, eye movements, channel pops, and drift) were manually removed. For each participant, signals free of artifacts were baseline corrected and averaged according to stimulus type, resulting in six ERPs per individual (three emotions [fearful, neutral, happy] across two sets [youth, adult]). The N170, P200, FN400, and P300 components were identified for each ERP. The N170 was quantified as the mean amplitude between 150 and 200 ms post-stimulus at P7 and P8 [49]. P200 was calculated as the mean amplitude from 190 to 230 ms post-stimulus at Pz [50], P300 from 270 to 330 ms post-stimulus at Pz [51], and FN400 from 300 to 500 ms post-stimulus at Fz [52].

### 2.6. ERP Paradigm

The emotional faces ERP paradigm was implemented using E-Prime 2.0, and consisted of 180 sequential trials. This setup included 30 child and adolescent faces (10 fearful, 10 neutral, and 10 happy) sourced from the National Institute of Mental Health Child Emotional Faces Picture Set (NIMH-ChEF) [53] and 30 adult faces (10 fearful, 10 neutral, and 10 happy) taken from the NimStim Set of Facial Expressions [54]. Faces were matched for sex and race/ethnicity (Caucasian, African-American, and Hispanic). The final stimulus set comprised 60 grayscale images of male and female faces spanning children, adolescents, and adults, with neutral, fearful, or happy expressions. Each trial began with a fixation cross displayed at the center of a computer screen for 500 ms, immediately followed by a face image shown for 1000 ms. Faces measuring 17 cm in height and 15 cm in width were displayed on a 27-inch flat-screen monitor. Participants sat in a comfortable chair approximately 34 cm from the screen and were instructed to observe each picture passively while keeping their gaze on the fixation cross. Across the 180 trials, each face appeared three times, with the presentation order randomized to ensure that no image was shown consecutively.

### 2.7. Neuropsychological Assessment

Participants completed a selection of subtests from the Cambridge Neuropsychological Test Automated Battery (CANTAB), a widely recognized computerized tool for assessing cognitive function. The study focused on cognitive domains relevant to affective information processing, comprehension and learning, decision-making, attention, and problem-solving, as these have been linked to cognitive changes in youth with PBD [4,5,6,8]. A summary of the tests evaluating these domains is outlined below.

The Affective Go/No-Go (AGN) test evaluates the biases in processing positive and negative information. Participants complete several blocks where they are shown words from three affective categories: positive, negative, and neutral. They must respond to words matching a designated target category. Key measures include reaction times and commission errors for positive and negative stimuli.

The Big/Little Circle (BLC) test measures comprehension, learning, and reversal learning. Over 20 trials, the participants initially selected the smaller of the two circles. The task then reverses for another 20 trials, requiring participants to choose a larger circle. The primary measure is the participant’s ability to correctly adjust their responses.

The Cambridge Gambling Task (CGT) assesses decision-making and risk-taking in a non-learning context. Participants view a row of ten boxes divided into red and blue sections, with one box hiding a yellow token. They decide whether the token is in a red or blue box and bet the points accordingly. Starting with 100 points, participants choose the proportion of points to wager by adjusting a central circle displaying the current bet value. The outcomes include decision-making quality, deliberation time, risk adjustment, risk-taking, delay aversion, and the proportion of points wagered.

The Match to Sample Visual Search (MTS) test examines attention and visual search abilities. A complex pattern is displayed on the screen, followed by a brief delay. Participants then see multiple patterns around the screen, one of which matches the original pattern. They identify the matching pattern, with accuracy (percentage of correct choices) as the key outcome.

The Stockings of Cambridge (SOC) test assesses spatial planning and problem-solving skills. Participants view two displays featuring three stockings, each containing colored balls arranged in different patterns. The task is to rearrange the balls in the lower display to replicate the pattern in the upper display using the fewest possible moves. The outcomes include the average number of moves required to complete the task.

### 2.8. Behavioral Assessment

The severity of behavioral symptoms was assessed using the Young Mania Rating Scale).

MRS) to evaluate manic symptoms [55] and the 17-item Children’s Depression Rating Scale-Revised (CDRS-R) to measure depressive symptoms [56]. Aggression was measured using the Aggression Questionnaire (AQ) [57], a 29-item instrument comprising four subscales that evaluate different facets of aggression: anger, physical aggression, hostility, and verbal aggression.

Motivational systems were assessed using the Behavioral Inhibition Scale/Behavioral Approach Scale (BIS/BAS) [58], which includes subscales for drive, fun seeking, reward responsiveness, and behavioral inhibition.

Borderline traits were evaluated using the Borderline Personality Features Scale for Children (BPFS-C) [59], a 24-item tool divided into four subscales that assess domains such as affective instability, identity problems, negative relationships, and self-harm.

Family dynamics were examined using the Family Environment Scale (FES) [60], a measure with 10 subscales covering three broad areas: relationship dimensions, personal growth (or goal orientation) dimensions, and system maintenance dimensions. In this study, only the primary scores were analyzed.

Anxiety symptoms were measured using the Screen for Child Anxiety Related Disorders (SCARED) [61], a 41-item self-report questionnaire with five subscales. Four subscales correspond to the DSM-IV-TR conceptualizations of anxiety disorders: panic disorder, generalized anxiety disorder, separation anxiety disorder, and social anxiety. The fifth subscale, school anxiety, addresses the prevalent anxiety issues in childhood and adolescence.

### 2.9. Statistical Analyses

#### 2.9.1. Sample Size Considerations

The number of participants was estimated through statistical power analysis, a review of the literature, and according to the recruitment capabilities of the center. Regarding *t*-tests regarding neuropsychological, behavioral, and neuroanatomical measurements, sensitivity analysis using G*Power [62] suggested that a minimum number of 42 subjects (21 subjects per group) can reach a power of 1-ß = 0.80, to detect an effect size of δ = 0.8 (α = 0.05; two-tailed). A separate measurement was performed for EEG measurements. In this case, sensitivity analysis suggested that a minimum number of 4 subjects is required to obtain a power of 1-ß = 0.80 to detect an effect size of δ = 0.8 (α = 0.05; two-tailed; number of measurements = 6, correlation among repeated measurements = 0.5, and non-sphericity correctio *n* = 1).

#### 2.9.2. Demographics and Clinical Characteristics

Multiple *t*-tests for continuous variables (i.e., age, IQ) and chi-square tests were used for nominal variables (i.e., gender, race/ethnicity) to assess differences in sociodemographic characteristics between groups.

#### 2.9.3. Differences in Neuroanatomy, Neurophysiology, Neuropsychology and Behavior

Five separate repeated measures GLMs were used to assess differences in ERP mean amplitude between groups. In each GLM, Groups (2 levels: PBD, HC) were used as between subjects variables, and set (2 levels: youth, adult faces) and emotion (3 levels: fearful, neutral, happy expressions) were used as within-subjects variables. The mean amplitude of the N170 at P7 and P8, the mean amplitude of P200 and P300 at Pz, and the mean amplitude of FN400 at Fz were used as dependent variables. In each GLM, age, sex, and IQ were used as covariates.

When needed, within-subject effects were corrected for sphericity violations using the Greenhouse-Geisser algorithm. The main effects and interactions were Bonferroni Corrected (*p* = 0.05/5 = 0.01).

Multiple T-tests were performed to investigate differences in deep gray matter structure volumes, cortical thickness, and scores at neuropsychological and psychopathological tests between groups. In each t-test, the two diagnostic groups (i.e., PBD and HCs) were used as independent variables, and ICV-corrected deep gray matter volumes, cortical area thicknesses, and scores on individual neuropsychological and psychopathological tests were used as dependent variables. In each t-test, age, sex, and IQ were used as covariates. Bonferroni correction was applied for multiple comparisons. Specifically, the significance of the *p*-value was set at *p* = 0.0035 (*p* = 0.05/14) for deep gray matter structures. For cortical thickness, the significance of the *p*-value was set at *p* = 0.00073 (*p* = 0.05/68). For scores on psychopathological tests, the significance of the *p*-value was set at *p* = 0.0021 (*p* = 0.05/23). For neuropsychological test scores, the significance of the *p*-value was set at *p* = 0.0035 (*p* = 0.05/14).

#### 2.9.4. Relationship Among Neuroanatomy, Neurophysiology, Neuropsychology and Behavior

In order to investigate the bi-directional relationships between neurobiological, neuropsychological, and psychopathological measures in youth with PBD, canonical correlation analyses (CCAs) were performed. CCAs represent an approach that identifies the relationships between two sets of variables [63]. The results of the CCAs are correlated pairs of latent variates, which are independent and composed of weighted sums of the predictor variable that maximally correlate with the weighted sums of the criterion variable. The interpretation of what the latent variates represent and how they are related to each other can be determined by the weighted loadings of individual measures on the latent structure, much like principal component analysis.

In this study, variables differentiating PBD from HCs were entered into the CCA, and then two separate CCAs were conducted for each type of measure (psychopathology, neuropsychology). Bonferroni correction was used for multiple testing across the two CCAs, with the significance set at *p* = 0.025. For each significant CCA pair, signs of loadings were used in order to interpret how scores on individual measures related to the latent variates. Therefore, loadings indicate which aspect of neurocognition/psychopathology is captured in each analysis, the neurobiological characteristics with which they are associated, and the nature of the relationship between them. In this view, a positive value of a loading indicates higher scores on the individual measures, whereas a negative value indicates lower scores on individual measures. Only moderate-strong loadings (beyond −0.3 or 0.3) were considered.

## 3. Results

### 3.1. Sample

Two hundred and sixty-one subjects were initially screened. Sixty-seven subjects were selected after applying the inclusion/exclusion criteria. Four subjects were incapable of sitting continuously and performing CANTAB/EEG/MRI measurements because of their mood state and were excluded from the sample. An additional 17 subjects were excluded because they did not complete all assessments. Therefore, the final sample was composed of 46 subjects (23 with PBD and 23 HCs).

### 3.2. Demographics and Clinical Characteristics

Participants did not show differences in age, gender, IQ, race, or ethnicity (see Table 1).

### 3.3. Neurophysiology

**Figure 1:**
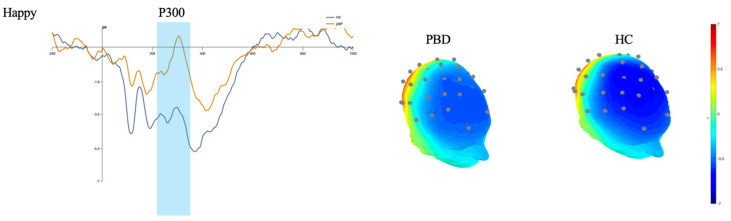
P300 amplitude for passive viewing of happy faces in subjects with PBD and HCs. (Left): waveform graph representing ERP data for happy stimuli; (right): without scalp topographies showing ERP activity for the P300 time window. Legend: HCs, healthy controls; PBD, pediatric bipolar disorder.

The mean ERP amplitudes at each electrode are reported in Table 2. A GLM investigating differences in P300 revealed a group-by-emotion interaction. Pairwise comparisons revealed that youth with PBD have greater P300 for happy faces than HCs (see Table 3 and Figure 1). Furthermore, in youth with PBD, happy faces elicited a higher P300 mean amplitude than fearful faces (see Table 4).

GLMs investigating differences in other ERPs did not show any significant differences between or within the groups.

### 3.4. Cortical Thickness

**Figure 2:**
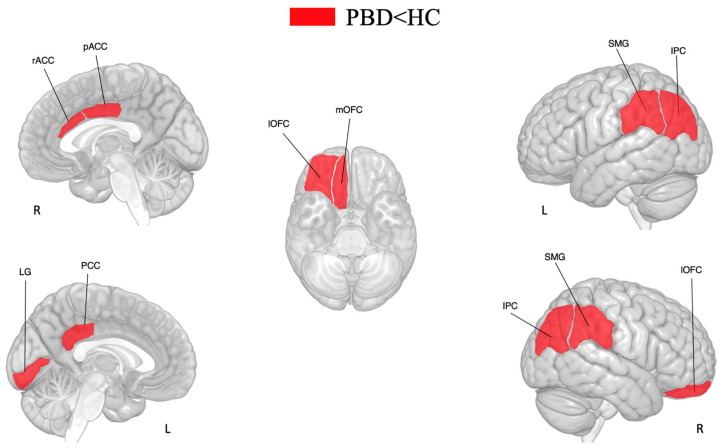
Differences in cortical thickness between subjects with PBD and HCs. The significant differences in cortical thickness are shown in red. Legend: HCs, healthy controls; IPC, inferior parietal cortex; SMG: supramarginal gyrus; LG, lingual gyrus; lOFC, lateral orbitofrontal cortex; mOFC, medial orbitofrontal cortex; pACC, posterior-anterior cingulate cortex; PBD, pediatric bipolar disorder; PCC, posterior cingulate cortex; rACC, rostral anterior cingulate cortex.

*T*-tests revealed that youth with PBD have reduced cortical thicknesses compared to HCs in the frontal (right rostral anterior cingulate, right caudal anterior cingulate, right lateral orbitofrontal, right medial orbitofrontal), parietal (right inferior parietal, left inferior parietal, left posterior cingulate, right supramarginal, left supramarginal), and occipital (left lingual) areas (see Table 5 and Figure 2).

### 3.5. Deep Gray Matter Structures

*T*-tests revealed no differences between the groups.

### 3.6. Neuropsychology

*T*-tests revealed significant differences between the groups in performance on the AGN and CGT tests. Specifically, regarding the AGN, youth with PBD committed more commission errors for negative stimuli compared to HCs. Regarding CGT, youth with PBD exhibited longer deliberation times and smaller risk adjustment scores than HCs (see Table 6).

### 3.7. Behavior

*T*-tests revealed significant differences between the PBD and HC groups in the CDRS-R, YMRS, SCARED, AQ, and BPFS-C. Specifically, youth with PBD exhibited higher scores on the CDRS-R and YMRS compared to HCs. Additionally, on the SCARED, they demonstrated higher total scores and elevated scores on the Separation Anxiety Disorder and Panic Disorder subscales. In the AQ, youth with PBD showed greater total scores and higher scores on the Anger and Hostility subscales than HCs. Similarly, in the BPFS-C, youth with PBD exhibited higher total scores and elevated scores on the negative relationship subscale compared to HCs (see Table 7).

### 3.8. Relationship Among Neuroanatomy, Neurophysiology, Neuropsychology and Behavior

Variables differentiating participants from HCs were three neurocognitive variables, seven psychopathological variables, and 12 neurobiological variables. The first CCA analysis included neurobiological variables in the first set and neuropsychological variables in the second set. The second CCA analysis included neurobiological variables as the first set and psychopathological variables as the second set. Age, sex, and IQ were entered into the model in the first set to evaluate the effects of such variables on psychopathological and neuropsychological performance. CCA analyses revealed that only the correlation between the first pair of variates for both models was significant (see Table 8). In the first CCA, IQ, P300 amplitude for happy faces, cortical thickness of the right lateral orbitofrontal, right rostral anterior cingulate, left inferior parietal, and left supramarginal thickness were positively correlated to the latent canonical variable, while age showed a significant negative correlation. Regarding neurocognitive measures, the number of commission errors for negative emotions in the AGN and deliberation time in the CGT was positively correlated with the latent canonical variate, whereas scores of risk adjustment in the CGT were negatively correlated.

**Figure 3:**
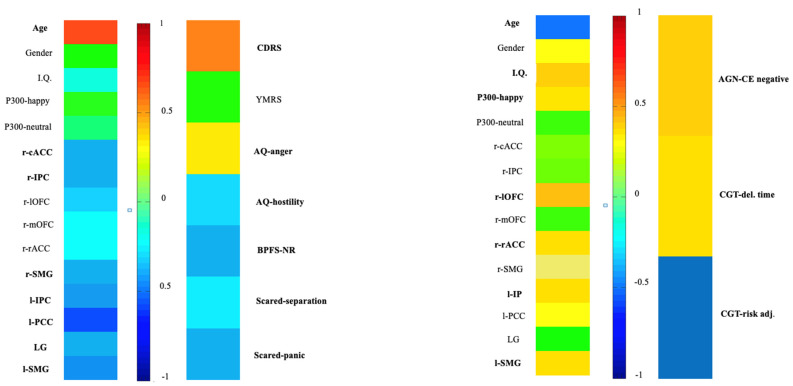
Relationship between predictors/dependent variables and canonical variables. (**Left**) loadings of the first CCA (neurobiological variables as the first set and neuropsychological variables as the second set); (**right**) loadings. Loadings are expressed in colors. Loadings > 0 are shown with worm colors, and loadings < 0 are shown with cold colors. Legend: cACC, caudal anterior cingulate cortex; CCA, canonical correlation analysis; HCs, healthy controls; IPC, inferior parietal cortex; LG, lingual gyrus; lOFC, lateral orbitofrontal cortex; mOFC, medial orbitofrontal cortex; PBD, pediatric bipolar disorder; PCC, posterior cingulate cortex; rACC, rostral anterior cingulate cortex; SMG, supramarginal gyrus.

In the second CCA, cortical thickness of the right caudal anterior cingulate, right inferior parietal, right supramarginal, left inferior parietal, left lingual, left posterior cingulate, and left supramarginal negatively correlated with the latent variate, while age positively correlated with it. Regarding psychopathological analyses, total scores of the CDRS-R positively correlated with the latent variate. Conversely, hostility scores of the AQ, scores of panic disorder and separation anxiety disorder subscales of the SCARED, and scores of the negative relationship subscale of the BPFS-C negatively correlated with this variate (See Figure 3).

## 4. Discussion

Youth with PBD showed higher P300 mean amplitude elicited by happy faces than fearful faces and cortical thinning in the frontal, parietal, and occipital areas than HCs. Additionally, they demonstrated a greater number of commission errors for negative stimuli on the AGN task, delayed deliberation times, and smaller risk adjustment scores on the CGT compared to HCs. On clinical assessments, youth with PBD reported elevated levels of manic, depressive, and anxiety symptoms; increased hostility and aggression; and more negative interpersonal relationships compared to HCs. In the PBD group, cortical thickening and P300 amplitude were positively correlated with higher commission errors for negative stimuli in the AGN task, longer deliberation times, reduced risk adjustment in the CGT, increased levels of panic and separation anxiety, and poorer interpersonal relationships. In contrast, these measures were negatively correlated with levels of depression. A recent systematic review showed an increased frequency of anxiety disorders such as separation anxiety, agoraphobia, generalized anxiety disorder, social phobia, and panic disorder in youth with PBD compared to the general population [64]. Previous studies also showed heightened anger, hostility, and interpersonal problems in youth with PBD, regardless of the phase of the disorder [65,66,67]. Our findings are also in line with the majority of studies comparing cortical thickness in youth with BD and HCs [20,21,68,69,70]. Nevertheless, the study of Zhang and Colleagues found alterations in the temporal cortex, whereas, in our study, no differences in this area emerged. Such a difference might be due to the age range of subjects recruited by Zhang and Colleagues, which is higher than ours. Since alterations in the temporal cortex have been found in subjects with adult BD [71], temporal thinning might add to the existing alterations at child/adolescent ages. Further studies are needed to clarify the cortical differences between youth with PBD and HCs.

The anterior cingulate and orbitofrontal cortices are part of the affective network [72,73,74] and play a role in the voluntary regulation of emotion [75]. These areas direct attentional resources to motivationally relevant stimuli and provide information about the emotional salience or significance of external stimuli in order to rapidly perceive reward contingency [76]. The parieto-occipital regions are part of a broader network involved in the initiation, maintenance, and control of goal-directed behavior, and are indirectly implicated in impulse control [77,78]. This network supports functions such as attention, working memory, response selection, response inhibition, and task switching, which are activated over time in order to overcome distraction and respond quickly to unpredictable demands that arise during performance [73]. Results from the AGN corroborate the evidence of difficulties in processing negative stimuli, whereas alterations found in the P300 amplitude are in contrast with what is expected. However, these findings are consistent with evidence from other late ERPs, such as LPP, and neuropsychological findings of heightened sensitivity to positively valenced stimuli [79,80,81]. Such positivity has been linked to the propensity to develop manic states [82]. Mismatches between findings from the AGN and those derived from the EEG might be related to the different stimuli used (words vs. faces). Language and facial expressions engage distinct areas of processing in the brain [83,84]. Furthermore, emotions used in the AGN, i.e., positive and negative words, are not completely comparable with those used in the ERP paradigm, i.e., happy versus fearful faces.

Results from the CGT regarding deliberation time and risk adjustment support the evidence of executive dysfunction in BD, especially in planning, strategy formation, and decision-making. Several studies have reported slower decision-making in individuals with BD than in HCs, both during manic episodes [80] and in the euthymic phase [81]. The literature provides strong evidence of significant deficits in strategy development and problem solving [85], which are present during acute episodes but may also persist during periods of remission [86].

Partially contrasting with expectations, CCAs showed a complex pattern of associations between neuroanatomical, neurophysiological, neurocognitive, and behavioral findings. Cortical thickening in areas associated with emotional regulation, decision-making, and control was linked to impairments in managing negative stimuli and prolonged decision-making times, while negatively correlating with difficulties in risk assessment. This finding aligns with evidence from functional MRI studies documenting heightened activity in the frontal, parietal, and occipital areas in emotional control [87] and, specifically, in response to negative stimuli [88]. Additionally, heightened negative stimuli correlated with greater P300 amplitude for happy faces might be the result of an effort to overcome prepotent impulse dyscontrol driven by negative emotions. Altered emotional processing might lead to the recruitment of cognitive resources, thereby reducing the cognitive resources dedicated to problem solving and prolonging deliberation times in the AGN. This alteration may also affect the possibility of recruiting resources to reassess risk, as demonstrated by the direct correlation between the aforementioned areas related to cognitive and emotional control, allocation of attentional resources toward positive stimuli, and risk adjustment in the CGT. Regarding the relationship found in the second CCA, the thickening of areas belonging to the fronto-parietal networks described above negatively correlates with levels of depression. Since the role of the affective network is to regulate mood, more significant alterations in areas within this network might lead to an increased severity of depressive states. Cortical thickening in these regions is also negatively correlated with the severity of hostility, panic symptoms, separation anxiety, and negative relationships. Nevertheless, hostile behaviors have been associated with manic states characterized by upregulated energy levels [89]. Similarly, borderline personality disorder traits, which include interpersonal difficulties [90], are characterized by high levels of energy, although dysregulated [91,92]. Disruption of high-energy levels has also been proposed as a possible mechanism for anxiety [93]. Furthermore, the consumption of substances that are supposed to increase body energy has been associated with the development of anxiety symptoms [94]. Consequently, cortical thickening of areas involved in mood regulation and levels of control might result in elevated energy levels. Such levels of energy may be dysregulated in those with PBD, potentially leading to increased anxiety, hostility, and behavioral dysregulation, which could contribute to interpersonal difficulties.

Limitations are as follows: The generalizability of the results is flawed by the small sample size. In addition, the small sample size limited the inclusion of other possible confounding variables, such as comorbidity and psychopharmacological treatments. Therefore, as previously mentioned, the present work should be corroborated by further research. Second, the cross-sectional design of the study limited the possibility of hypothesizing causal inferences among the dimensions measured. Therefore, longitudinal studies are needed to further investigate this interplay. Third, although the present study investigated a wide array of behavioral symptoms, certain variables should have been considered when investigating behavior in PBD. Specifically, the psychotropic medications used by subjects with PBD in this study were described as classes. Specific psychotropic medications have been shown to modify brain structure and function [94]. However, we were unable to study the effect of a single medication due to the small sample size. Therefore, additional studies are needed to evaluate the effects of psychotropic medications in patients with PBD. Other variables that should be investigated include the subjects’ affective temperament and predominant polarity [95,96]. Affective temperament refers to the temporally stable individual’s activity level, rhythms, moods, and related cognitions as well as their variability, whereas predominant polarity is the pattern of mood episodes that an individual experiences most frequently over a certain period [97]. Both concepts have been proven to influence mood brain structure and function [96,98]. Therefore, greater attention should be paid to these two concepts.

## 5. Conclusions

PBD is characterized by a wide array of behavioral, neuropsychological, neuroanatomical, and neurophysiological alterations compared to HCs. A complex relationship links all these dimensions. The foundations of this relationship include areas involved in mood, energy, and cognitive control; interpretations of positive and negative stimuli; and behavioral disturbances related to mood and energy imbalance. These preliminary findings corroborate the relationship between the brain and behavior. Furthermore, the findings suggest that research aimed at identifying neuromarkers of cognitive and behavioral alterations in PBD should be encouraged. Nevertheless, because of the limitations described above, this work should be considered preliminary. Additional studies with larger sample sizes are needed to confirm the present findings and define a comprehensive framework that incorporates brain-behavior relationships in PBD.

## Figures and Tables

**Table 1:** Sociodemographic and clinical characteristics of subjects with PBD and HCs.

			PBD (N = 23)	HC (N = 23)	F or χ2	*p*-Value
Demographics						
	Age (years), mean ± SD		12.26 ± 3.24	12.00 ± 3.25	0.07	0.791
	Female, n (%)		15 (65.22)	13 (56.50)	0.37	0.553
	Race, n (%)					
		*Asian*	0 (0.00)	3 (13.04)		
		*African-American*	2 (8.69)	4 (17.39)	4.34	0.112
		*Caucasian*	21 (91.30)	16 (69.56)		
	Ethnicity, n (%)					
		*Hispanic*	5 (21.73)	1 (4.34)	3.07	0.084
	IQ, mean ± SD		103.22 ± 13.36	98.61 ± 15.70	1.15	0.296
Clinical						
	Year ill (years), mean ± SD		3.30 ± 2.30	-	-	-
	PBD type, n (%)					
		*Type I*	15 (65.22)	-	-	-
		*Type II*	1 (4.35)			
		*Not otherwise specified*	7 (30.43)	-	-	-
	Comorbidity, n (%)					
		*None*	10 (43.48)	-	-	-
		*ADHD*	7 (30.43)	-	-	-
		*OCD*	1 (4.35)	-	-	-
		*Panic Disorder*	1 (4.35)	-	-	-
		*Generalized Anxiety Disorder*	4 (17.39)	-	-	-
		Mood state, n (%)				
		*Euthymic*	10 (43.48)	-	-	-
		*Manic/hypomanic*	7 (30.44)	-	-	-
		*Depressed*	3 (13.04)	-	-	-
		*Depressed with mixed features*	3 (13.04)	-	-	-
	Current pharmacotherapy, n (%)					
		*AD*	12 (52.18)	-	-	-
		*AP*	10 (43.48)	-	-	-
		*MS*	11 (47.82)	-	-	-
		*BDZ*	0 (0.00)	-	-	-
		*MARI*	8 (34.78)	-	-	-

AD, antidepressant; ADHD, attention-deficit hyperactivity disorder; AP, antipsychotic; BDZ, benzodiazepine; HCs, healthy controls; IQ, intelligence quotient; MARI, mixed monoamine reuptake inhibitor; MS, mood stabilizer; SD, standard deviation; OCD, obsessive-compulsive disorder; PBD, pediatric bipolar disorder.

**Table 2:** ERP amplitudes for happy faces in subjects with PBD and HCs.

			PBD (N = 23)	HC (N = 23)
ERP				
	N170 (P7)			
		*Fearful, μV, mean* ± SD	1.30 ± 3.47	0.62 ± 2.47
		*Neutral, μV, mean* ± SD	1.41 ± 3.31	0.05 ± 1.95
		*Happy, μV, mean* ± SD	1.87 ± 3.10	0.41 ± 2.43
	N170 (P8)			
		*Fearful, μV, mean* ± SD	2.87 ± 3.84	2.23 ± 4.02
		*Neutral, μV, mean* ± SD	2.07 ±3.26	2.12 ± 3.39
		*Happy, μV, mean* ± SD	3.07 ± 3.88	2.08 ± 3.99
	P200 (Pz)			
		*Fearful, μV, mean* ± SD	−1.72 ± 5.78	−0.31 ± 5.56
		*Neutral, μV, mean* ± SD	−1.93 ± 3.40	−2.45 ± 3.53
		*Happy, μV, mean* ± SD	−1.40 ± 5.30	−3.15 ± 4.07
	FN400 (Fz)			
		*Fearful, μV, mean* ± SD	−10.09 ± 5.56	−8.65 ± 7.86
		*Neutral, μV, mean* ± SD	−8.75 ± 4.69	−8.65 ± 7.86
		*Happy, μV, mean* ± SD	−8.84 ± 5.29	−9.97 ± 5.64
	P300 (Pz)			
		*Fearful, μV, mean* ± SD	−1.88 ± 5.35	−0.95 ± 5.03
		*Neutral, μV, mean* ± SD	0.47 ± 5.33	−2.80 ± 4.55
		*Happy, μV, mean* ± SD	0.95 ± 10.66	−5.60 ± 9.11

Legend: ERP, event-related potential; SD, standard deviation.

**Table 3:** Differences between subjects with PBD and HCs in mean P300 mean amplitude.

	PBD		HC	
	Mean Diff	*p*-Value	Mean Diff	*p*-Value
***Happy* vs. *fearful, μV, mean* ± SD**	1.64	**0.007**	1.85	**0.** **046**
*Neutral* vs. *fearful, μV, mean* ± SD	2.36	0.061	1.56	0.081
*Happy* vs. *neutral, μV, mean* ± SD	0.71	0.793	0.288	>0.999

Legend: significant results are in **bold**. SD: standard deviation.

**Table 4:** Differences in mean p300 amplitude to emotional faces in subjects with PBD and HCs.

Faces at Pz	PBD vs. HC	
	**F**	***p*-Value**
*Fearfull, μV, mean* ± SD	0.33	0.569
*Neutral, μV, mean* ± SD	0.06	0.085
***Happy, μV, mean* ± SD**	**5.13**	**0.029**

Legend: significant results are in **bold**. Diff, difference; HCs, healthy controls; PBD, pediatric bipolar disorder; SD, standard deviation.

**Table 5:** Cortical thickness in subjects with PBD and HCs.

		PBD (N = 23)	HC (N = 23)		*F*	*p*-Value
Cortical areas						
	Frontal					
		***R Rostral anterior cingulate***	3.10 ± 0.27	3.16 ± 0.35	8.69	**<0.001**
		*L Rostral anterior cingulate*	2.99 ± 0.29	3.09 ± 0.24	2.59	0.051
		***R Caudal anterior cingulate***	2.62 ± 0.22	2.78 ± 0.24	12.63	**<0.001**
		*L Caudal anterior cingulate*	2.78 ± 0.33	2.77 ± 0.11	3.11	0.025
		*R Isthmus cingulate*	2.55 ± 0.16	2.64 ± 0.18	2.69	0.044
		*L Isthmus cingulate*	2.53 ± 0.16	2.61 ± 0.21	2.76	0.040
		*R Caudal middle frontal*	2.61 ± 0.14	2.66 ± 0.12	2.22	0.126
		*L Caudal middle frontal*	2.62 ± 0.13	2.64 ± 0.09	1.70	0.168
		***R Lateral orbitofrontal***	2.78 ± 0.20	2.89 ± 0.17	8.77	**<0.001**
		*L Lateral orbitofrontal*	2.85 ± 0.22	2.25 ± 0.12	4.02	0.008
		***R Medial orbitofrontal***	2.61 ± 0.20	2.70 ± 0.23	7.29	**<0.001**
		*L Medial orbitofrontal*	2.59 ± 0.26	2.60 ± 0.19	3.44	0.016
		*R Pars opercularis*	2.76 ± 0.18	2.64 ± 0.17	2.82	0.037
		*L Pars opercularis*	2.80 ± 0.17	2.83 ± 0.12	0.883	0.132
		*R Pars orbitalis*	2.86 ± 0.24	2.96 ± 0.20	2.24	0.082
		*L Pars orbitalis*	2.91 ± 0.25	2.98 ± 0.19	1.54	0.208
		*R Pars triangularis*	2.65 ± 0.21	2.63 ± 0.18	1.87	0.134
		*L Pars triangularis*	2.64 ± 0.17	2.69 ± 0.14	1.86	0.136
		*R Precentral*	2.54 ± 0.18	2.66 ± 0.10	2.15	0.092
		*L Precentral*	5.13 ± 0.30	5.36 ± 0.17	2.47	0.059
		*R Rostral middlefrontal*	2.49 ± 0.18	2.48 ± 0.12	3.97	0.008
		*L Rostral middlefrontal*	2.47 ± 0.17	2.46 ± 0.11	5.07	0.002
		*R Superior frontal*	2.85 ± 0.20	2.93 ± 0.08	3.90	0.009
		*L Superior frontal*	2.83 ± 0.18	2.88 ± 0.13	3.84	0.010
		*R Frontal pole*	2.89 ± 0.32	2.90 ± 0.33	1.81	0.053
		*L Frontal pole*	2.89 ± 0.32	2.81 ± 0.34	2.16	0.090
	Temporal					
		*R Bank STS*	2.75 ± 0.24	2.87 ± 0.20	2.68	0.045
		*L Bank STS*	2.77 ± 0.19	2.77 ± 0.11	2.11	0.097
		*R Entorhinal*	3.45 ± 0.47	3.43 ± 0.41	0.73	0.577
		*L Entorhinal*	3.36 ± 0.37	3.48 ± 0.33	1.94	0.122
		*R Fusiform*	2.86 ± 0.20	2.92 ± 0.13	2.38	0.067
		*L Fusiform*	2.91 ± 0.17	3.00 ± 0.13	3.97	0.008
		*R Superior temporal*	2.99 ± 0.25	3.10 ± 0.20	2.79	0.039
		*L Superior temporal*	2.93 ± 0.25	3.04 ± 0.12	2.01	0.111
		*R Middle temporal*	3.08 ± 0.24	3.15 ± 0.12	1.73	0.161
		*L Middle temporal*	2.99 ± 0.24	3.11 ± 0.16	2.14	0.094
		*R Inferior temporal*	2.88 ± 0.20	2.88 ± 0.12	1.83	0.141
		*L Inferior temporal*	2.88 ± 0.22	2.96 ± 0.14	1.68	0.199
		*R parahippocampal*	2.90 ± 0.25	3.00 ± 0.21	2.19	0.087
		*L parahippocampal*	2.99 ± 0.32	3.08 ± 0.29	1.88	0.132
		*R Temporal pole*	3.71 ± 0.31	3.77 ± 0.33	0.69	0.604
		*L Temporal pole*	3.64 ± 0.35	3.80 ± 0.28	0.82	0.518
		*R Transversal temporal*	2.70 ± 0.30	2.84 ± 0.24	2.39	0.066
		*L Transversal temporal*	2.70 ± 0.26	2.83 ± 0.23	5.87	0.001
		*R Insula*	3.17 ± 0.21	3.22 ± 0.19	3.21	0.022
		*L Insula*	3.21 ± 0.16	3.30 ± 0.21	2.70	0.044
	Parietal					
		*R Superior parietal*	2.22 ± 0.14	2.29 ± 0.13	4.64	0.004
		*L Superior parietal*	2.26 ± 0.16	2.31 ± 0.13	4.07	0.007
		***R Inferior parietal***	2.58 ± 0.13	2.62 ± 0.15	12.05	**<0.001**
		***L Inferior parietal***	2.56 ± 0.20	2.58 ± 0.16	6.27	**<0.001**
		*R Paracentral*	2.50 ± 0.22	2.64 ± 0.17	1.98	0.116
		*L Paracentral*	2.52 ± 0.20	2.63 ± 0.18	3.18	0.025
		*R Posterior cingulate*	2.64 ± 0.19	2.71 ± 1.00	2.87	0.035
		***L Posterior cingulate***	2.65 ± 0.21	2.74 ± 0.17	6.49	**<0.001**
		*R Postcentral*	2.18 ± 0.17	2.21 ± 0.18	2.83	0.010
		*L Postcentral*	5.13 ± 0.30	5.36 ± 0.17	2.35	0.070
		*R Precuneus*	2.56 ± 0.16	2.61 ± 0.12	5.16	0.002
		*L Precuneus*	2.56 ± 0.16	2.60 ± 0.14	11.33	0.001
		***R Supramarginal***	2.70 ± 0.14	2.77 ± 0.14	11.01	**<0.001**
		***L Supramarginal***	2.71 ± 0.15	2.82 ± 0.15	8.82	**<0.001**
	Occipital					
		R Cuneus	2.01 ± 0.14	2.10 ± 0.17	4.09	0.007
		*L Cuneus*	2.03 ± 0.19	2.03 ± 0.15	5.37	0.001
		*R lateral occipital*	2.27 ± 0.13	2.32 ± 0.12	1.92	0.126
		*L lateral occipital*	2.22 ± 0.15	2.25 ± 0.12	2.93	0.032
		*R lingual*	2.21 ± 0.15	2.24 ± 0.16	5.38	0.001
		***L lingual***	2.18 ± 0.17	2.21 ± 0.14	8.15	**<0.001**
		*R pericalcarine*	1.75 ± 0.15	1.79 ± 0.09	1.71	0.166
		*L pericalcarine*	1.80 ± 0.20	1.78 ± 0.13	1.25	0.305

Legend: significant results are in **bold**. HCs, healthy controls; L, left; PBD, pediatric bipolar disorder; R, right; SD, standard deviation.

**Table 6:** CANTAB tests in subjects with PBD and HCs.

			PBD (N = 23)	HC (N = 23)	F	*p*-Value
Test						
	Affective Go/No-go					
		*RT positive stimuli (s)* mean ± SD	542.58 ± 137.92	483.35 ± 129.15	0.84	0.440
		*RT negative stimuli (s)* mean ± SD	525.71 ± 118.85	481.33 ± 505.99	0.80	0.457
		*CE positive stimuli (n)* mean ± SD	12.00 ± 8.51	11.00 ± 8.72	5.33	0.010
		***CE negative stimuli (n)* mean** **± SD**	13.25 ± 10.62	11.37 ± 8.29	7.15	**0.003**
	Cambridge gambling task					
		*Delay aversion*, mean ± SD	0.55 ± 0.29	0.59 ± 23	1.58	0.217
		***Deliberation time (ms)***, mean ± SD	384.13 ± 2112.22	252.51 ± 1115.99	12.34	**<0.001**
		*Overall proportion bet*, mean ± SD	0.55 ± 0.17	0.53 ± 0.12	0.25	0.782
		*Quality of decision making*, mean ± SD	0.90 ± 0.11	0.84 ± 0.16	1.51	0.234
		***Risk adjustment,*** mean ± SD	0.18 ± 1.02	0.51 ± 1.02	7.08	**0.002**
		*Risk taking*, mean ± SD	0.58 ± 0.17	0.56 ± 0.14	0.14	0.870
	Stockings of Cambridge					
		*Problem solved in minimum moves (n), mean* ± SD	5.65 ± 1.82	6.30 ± 152.03	6.51	0.004
	Spatial Recognition Memory					
		*% of correct trials, mean* ± SD	65.00 ± 13.48	75.71 ± 12.68	6.48	0.008
	Big/Little Circle					
		*% of correct selection, mean* ± SD	97.05 ± 3.30	96.74 ± 3.49	0.17	0.845
	Match to Simple Visual Search					
		*% of correct choice, mean* ± SD	97.02 ± 3.82	96.13 ± 4.02	0.50	0.614

Legend: significant results are in **bold**. CE, commission error; HCs, healthy controls; L, left; PBD, pediatric bipolar disorder; R, right; RT, reaction time; SD, standard deviation; s, seconds.

**Table 7:** Significance of CCA variate pairs in subjects with PBD.

CCA	Canonical Correlation	Squared Canonical Correlation	Eigenvalue	Wilk’s Lambda	F	*p*-Value
Neuropsychology						
**Pair1**	0.98	0.96	25.72	0.001	3.19	0.008
Psychopathology						
**Pair1**	0.99	0.99	63.53	<0.001	3.53	0.003

Legend: significant results are in **bold.** CCA: canonical correlation analysis; PBD: pediatric bipolar disorder.

**Table 8:** Psychopathological scales in subjects with PBD and HCs.

			PBD (N = 23)	HC (N = 23)	F	*p*-Value
Test						
	**Children’s Depression Rating Scale-Revised, mean ± SD**		**33.96 ± 13.75**	**17.39 ± 4.11**	**7.76**	**<0.001**
	**Young Mania Rating Scale, mean ± SD**		**13.83 ± 10.16**	**2,70 ± 3.43**	**5.09**	**0.001**
	Family Environment Scale, mean ± SD		47.04 ± 3.90	44.09 ± 13.08	1.11	0.364
	Screen for Child Anxiety Related Disorders					
		**Panic disorder, mean ± SD**	**7.57 ± 5.14**	**2.09 ± 3.69**	**5.47**	**0.001**
		Generalized anxiety disorder, mean ± SD	8.04 ± 5.17	3.13 ± 4.24	4.05	0.004
		**Separation anxiety disorder, mean ± SD**	**6.35 ± 3.99**	**2.04 ± 2.48**	**11.00**	**<0.001**
		Social anxiety, mean ± SD	2.35 ± 2.33	0.70 ± 0.77	4.31	0.005
		School anxiety, mean ± SD	4.70 ± 2.74	3.70 ± 2.65	1.14	0.353
		**Total, mean ± SD**	**29.09 ± 14.41**	**11.74 ± 11.18**	**7.35**	**<0.001**
	Aggression Questionnaire					
		**Anger, mean ± SD**	**19.35 ± 6.30**	**12.39 ± 3.59**	**7.27**	**<0.001**
		Physical aggression, mean ± SD	20.00 ± 7.94	14.52 ± 5.11	2.54	0.055
		**Hostility, mean ± SD**	**20.30 ± 7.60**	**11.13 ± 5.55**	**6.23**	**0.001**
		Verbal aggression, mean ± SD	13.61 ± 4.71	10.22 ± 5.43	3.47	0.016
		**Total, mean ± SD**	**29.09 ± 14.41**	**11.74 ± 11.18**	**6.55**	**<0.001**
	Behavioral Inhibition Scale/Behavioral Avoidance Scale					
		Drive, mean ± SD	8.30 ± 2.54	11.09 ± 3.68	12.48	0.005
		Fun seeking, mean ± SD	7.87 ± 1.25	8.65 ± 1.53	2.90	0.096
		Reward responsiveness, mean ± SD	9.74 ± 2.75	10.09 ± 3.26	9.98	0.014
		Behavioral inhibition, mean ± SD	15.22 ± 3.19	16.13 ± 2.34	1.78	0.189
	Borderline Personality Features Scale-Child					
		Affective instability, mean ± SD	17.22 ± 3.19	16.13 ± 2.34	7.59	0.009
		Identity problems, mean ± SD	15.13 ± 6.51	10.52 ± 6.51	7.74	0.008
		Self-harm, mean ± SD	15.91 ± 5.76	11.65 ± 3.79	9.06	0.004
		**Negative relationship, mean ± SD**	**15.17 ± 4.62**	**10.57 ± 3.09**	**16.52**	**<0.001**
		**Total, mean ± SD**	**68.08 ± 17.18**	**44.96 ± 9.81**	**13.37**	**0.001**

Legend: significant results are in **bold**. HCs, healthy controls; PBD, pediatric bipolar disorder; SD, standard deviation.

## Data Availability

Data are available upon request.
